# Hypoxemia and its clinical predictors among children with respiratory distress admitted to the University of Gondar Comprehensive Specialized Hospital, Northwest Ethiopia

**DOI:** 10.1186/s12887-024-04892-y

**Published:** 2024-06-27

**Authors:** Deresse Gugsa Tamene, Alemayehu Teklu Toni, Mohammed Seid Ali

**Affiliations:** 1 Department of Pediatrics and Child Health, School of Medicine, College of Medicine and Health Sciences, University of Gondar, Gondar, Ethiopia; 2 Department of Pediatrics and Child Health Nursing, School of Nursing, College of Medicine and Health Sciences, University of Gondar, Gondar, Ethiopia

**Keywords:** Hypoxemia, Respiratory distress, Clinical predictors, Children, Ethiopia

## Abstract

**Introduction:**

Hypoxemia is a common complication of childhood respiratory tract infections and non-respiratory infections. Hypoxemic children have a five-fold increased risk of death compared to children without hypoxemia. In addition, there is limited evidence about hypoxemia and clinical predictors in Ethiopia. Therefore, this study was conducted to assess the prevalence and clinical predictors of hypoxemia among children with respiratory distress admitted to the University of Gondar Comprehensive Specialized Hospital.

**Methods:**

An institutional-based cross-sectional study was conducted from December 2020 to May 2021 in northwest Ethiopia. A total of 399 study participants were selected using systematic random sampling. The oxygen saturation of the child was measured using Masimo rad-5 pulse oximetry. SPSS version 21 software was used for statistical analysis.

**Result:**

In this study, the prevalence of hypoxemia among children with respiratory distress was 63.5%. The clinical signs and symptoms significantly associated with hypoxemia were: head-nodding (AOR: 4.1, 95% CI: 1.81–9.28) and chest indrawing (AOR: 3.08, 95% CI: 1.32–7.16) which were considered statistically the risk factors for hypoxemia while inability to feed (AOR: 0.13, 95% CI: 0.02–0.77) was the protective factor for hypoxemia. The most sensitive predictors of hypoxemia were fast breathing with sensitivity (98.4%), nasal flaring (100.0%), chest indrawing (83.6%), and intercostal retraction (93.1%). The best specific predictors of hypoxemia were breathing difficulty with specificity (79.4%), inability to feed (100.0%), wheezing (83.0%), cyanosis (98.6%), impaired consciousness (94.2%), head-nodding (88.7%), and supra-sternal retraction (96.5%).

**Conclusion and recommendation:**

The prevalence of hypoxemia among children was high. The predictors of hypoxemia were the inability to feed, head nodding, and chest indrawing. It is recommended that the health care settings provide immediate care for the children with an inability to feed, head nodding, and chest indrawing. The policymakers better to focus on preventive strategies, particularly those with the most specific clinical predictors.

## Introduction

Hypoxemia is defined as a condition where arterial oxygen tension (Pao2) is below normal (normal Pao2 = 80–100 mmHg) and oxygen saturation is less than 90% [1]. Hypoxemia is a common complication of childhood infections, particularly respiratory tract infections. Among respiratory infections, pneumonia disproportionately impacts developing countries and accounts for more than two million child deaths worldwide. In addition to respiratory tract infections, hypoxemia also occurs in severe sepsis, meningitis, common neonatal problems, and other conditions that impair ventilation and gas exchange or increase oxygen demands. This could be mainly due to the low accuracy of clinical predictors and the limited availability of pulse oximetry for more accurate detection and oxygen for treatment [2]. Hypoxemia is a common complication of treatable, fatal childhood illnesses, and the treatment of hypoxemia with oxygen is a priority of WHO Integrated Management of Childhood Illness (IMCI) and Emergency Triage Assessment and Treatment (ETAT) guidelines [3].

In Africa, hypoxemia is a potentially harmful complication of both acute lower respiratory tract infections and non-respiratory tract infections in children, but its contribution to the burden and outcomes of hospital admissions is unclear [4]. Children who suffer from hypoxemia in lower acute respiratory infections have a five-fold increased risk of death compared to children without hypoxemia. Because of this impact, early detection of hypoxemia is a very crucial step to prevent its complications [5]. Respiratory failure is the most common cause of cardiopulmonary arrest in the pediatric population; therefore, it is important for emergency providers to recognize respiratory distress quickly in children of all ages and intervene aggressively to prevent respiratory failure [6].

The previous studies showed that the prevalence of hypoxemia in India was 23.8 [7]. Another study was conducted in India 39.7% [8] and 48% [9] and in Nepal 37% [10] and 51% [11]. A case-control study was conducted in Indonesia to identify clinical predictors of hypoxemia in severely malnourished children under five with pneumonia, the magnitude of hypoxemia among cases was 30% and among controls 4% [6]. In another study conducted among those aged greater than two months in Indonesia, the prevalence of hypoxemia was 41.6% [12].

The clinical signs and symptoms associated with hypoxemia identified in some previous studies showed that cyanosis, head nodding, drowsiness, fast respiratory rate for age, lower chest wall indrawing, grunting, the absence of crying during the examination, and the inability to breastfeed or drink were good predictors of hypoxemia [12, 13]. No single sign is a reliable predictor, and sensitivity is generally low for any single sign. There was also a study conducted in rural Zambia that showed that the clinical predictors of hypoxemia among children with respiratory distress were tachypnea and crepitation’s/bronchial breathing [14]. Most of the previous studies were conducted among a narrow range of pediatric age groups and mainly focused on lower respiratory infections.

In Ethiopia, according to a study conducted in 14 hospitals among children with respiratory distress, the prevalence of hypoxia was 57.0% [15]. Currently, many Ethiopian health centers do not have fully functional oxygen cylinders, concentrators, or oximetry available. Standard operating procedures or job aids for safe and effective oxygen therapy or identification of children with hypoxemia or severe disease are not common, and often inadequate staff is trained in providing oxygen therapy [16]. However, Ethiopia is addressing this situation head-on. One previous study conducted in Ethiopia in 14 hospitals showed that the prevalence of hypoxemia was 57.0%. To increase access to oxygen and pulse oximetry, the Ethiopian Ministry of Health (MoH) has responded by undertaking a comprehensive strategic planning process captured as a roadmap for oxygen and pulse oximetry scale-up [15]. Despite efforts being made to address hypoxemia and its associated mortality, more needs to be done. Children in respiratory distress will continue to die without access to pulse oximetry, oxygen, or inadequately trained staff. Effective oxygen therapy requires prompt and accurate detection of hypoxemia and appropriate administration of oxygen, combined with good clinical evaluation and management of the underlying condition. Early identification of hypoxemia with pulse oximetry and appropriate treatment with oxygen is lifesaving, yet availability and access to both services remain inadequate in low-resource settings. In developing countries like Ethiopia, identifying the clinical predictors of hypoxemia is crucial for management and priority setting, particularly in health centers and primary hospitals. A fundamental step in the prevention and control of hypoxemia is the identification of clinical predictors contributing to the rapidly increasing rate of the problem. There were no studies conducted to assess the prevalence of hypoxemia and its clinical predictors among children admitted with respiratory distress in Ethiopia, particularly in the study area.

Therefore, this study was conducted to determine the prevalence and clinical predictors of hypoxemia among children admitted with respiratory distress to the University of Gondar Comprehensive Specialized Hospital. As a result, this study will have a great contribution to the design of preventive action and the management of hypoxemia and its associated health problems.

## Methods

### Study area and period

An institutional-based cross-sectional study design was conducted from December 2020 to May 2021 among children with respiratory distress admitted to Gondar University Comprehensive Specialized Hospital. The hospital is located 748 km away from Addis Ababa. This hospital is located in Gondar city in the central Gondar Zone, which is in the Amhara region and located in the northwest part of Ethiopia.

#### Study participants

The participants were children admitted with respiratory distress in the pediatric emergency ward at the University of Gondar Comprehensive Specialized Hospital. Sick children admitted with respiratory distress and aged between 1 month and 18 years were included in the study. Children with chronic respiratory illnesses (broncho-pulmonary dysplasia, cystic fibrosis, and lung malformations), cardiopulmonary resuscitation in the past, patients with carbon monoxide poisoning, CNS malformations, and neuromuscular disorders were excluded from the study.

### Sample size determination

The sample size for this study was estimated by two methods for the two specific objectives; For the first objective, we applied a single population proportion formula. For the second objective, the sample size determination was tested by clinical predictors and was very small, so we omitted it. Lastly, the larger sample size was taken, and the first sample size was determined by using a single population proportion formula by considering the following statistical assumptions: 95% CI, taking the prevalence of hypoxemia, *p* = 57.0% from the previous study conducted in Ethiopia in 14 hospitals [17]. By adding a 5% non-response rate, the final sample size was 399.

### Sampling procedures

A systematic random sampling technique was used to select 399 participants from children admitted to the pediatric emergency ward. The total estimated cases per 4 months were approximately 800 (every 2 patients were selected: k = N/n, i.e., k = 800/399 = 2.005). Where N = total cases and n = calculated sample size. The participants were selected based on the total number of estimated patients admitted with respiratory distress in pediatric emergencies.

### Variables of the study

This study included dependent and independent variables. The dependent variable was hypoxemia, and independent clinical predictors were age of child, sex of child, inability to feed or drink, diarrhea, vomiting, seizure, nasal flaring, costal retraction cyanosis, chest in drawing, head nodding, grunting, crepitation, and wheezing. The outcome variable was dichotomized into “hypoxemic” when the arterial oxygen saturation measured by pulse oximetry is below 90% and “non-hypoxemic” when the arterial oxygen saturation measured by pulse oximetry is above 90%.

### Operational definition

#### Hypoxemia

when the arterial oxygen saturation with pulse oximetry is below 90%.

#### Cyanosis

a condition in which the red cells in the blood are not fully loaded with oxygen and the skin and mucous membranes appear blue.

#### Nutritional status

measured by BMI (Body Mass Index) for age z-score and described as: normal if z-score between + 1 and − 1; undernourished if z-score <-2; and overnourished if z-score > + 1.

#### Respiratory distress

signs and symptoms of an abnormal respiratory pattern.

#### Breathing difficulty

when the patient manifests an uncomfortable feeling of not being able to breathe well enough.

#### Fast breathing

breathing rate per minute higher than normal for the age group category; greater than 60 breaths/minute for infants aged less than 2 months, greater than 50 breaths/minute for infants aged 2 months to 12 months, greater than 40 breath/min for the age category of 12 months to 5 years, and greater than 30 for children aged 5 years or more.

#### Cyanosis

a bluish discoloration of the skin and mucous membrane due to a low oxygen level in the tissue or blood.

#### Mental status

a state of the patient’s general awareness, cognitive function, and responsiveness; it might be conscious and alert, unconscious, lethargic, stupor, or comatose.

#### Anthropometric measurement

the use of specific tools to collect the data needed to assess the health and growth status of the human body, such as weight, height, and middle upper arm circumference.

#### Impaired consciousness

any change from complete self-awareness to inhibit or absent self-awareness and arousal.

### Data collection (tools, procedures, and quality control)

A structured, pretested, and interviewer-administered questionnaire was used to collect the data. The questionnaire was adapted from the previous study [17], and it contains socio-demographic factors, clinical symptoms, and signs of respiratory distress in children. Some clinical signs and symptoms were reviewed from the patient chart. Finally, the arterial oxygen saturation of the child was measured by general physicians and residents using Massimo Rad 5 pulse-oximetry after the finger was cleaned and dried, and anthropometric measurements of weight and height of the children were collected. To collect an accurate measurement of the oxygen saturation, it was measured in emergency room during admission before starting any oxygen therapy. Data were collected by three general practitioners and two residents. The data collection procedures were supervised by the investigator and one resident.

### Data quality control

Data collectors and supervisors were trained for one day about the whole procedure of data collection to minimize errors. The questionnaire was developed in English and will be checked by language experts for consistency of the language, and it will be reviewed by a physician to check its appropriateness for assessing hypoxemia and its clinical predictors in children with respiratory distress. The data were collected after a pretest was conducted on 5% of children admitted with respiratory distress in the pediatric emergency ward other than those included in the actual study. The principal investigator and supervisors had day-to-day onsite supervision during the whole period of data collection. The collected data were reviewed and checked for completeness, accuracy, and consistency by supervisors and investigators. The oxygen saturation measurement (pulse oximetry) machine was checked by biomedical technicians for its accuracy of measurement. The completeness of the questionnaire was checked before data entry, too. The questioner was anonymous, and the collected data was stored in a confidential place.

### Data processing and analysis

Data were first coded and entered using EPI Info version 7.1 for data exploration and cleaning. The cleaned data were exported to SPSS version 20 statistical packages for statistical analysis. Descriptive and analytic statistics were carried out for the variables. The magnitude of hypoxemia was determined descriptively. A bivariable analysis of signs and symptoms predicting hypoxemia was done first, and then a *p*-value < 0.2 was checked by multivariable logistic regression analysis to check the independent utility of these signs and symptoms. A binary logistic regression model was fitted to identify the factors associated with the outcome variable. For this study, the analysis model was fitted and tested. The model’s fitness was tested by Hosmer and Lemeshow goodness of fit; analyses of the results were done in the form of sensitivity, specificity, Positive Predictive Value (PPV), and Negative Predictive Value (NPV). A Chi-square test was applied to compare the signs between hypoxemic and non-hypoxemic children. The odds ratio with a 95% confidence interval was computed to assess the level of association and direction of association, as well as the statistical significance. A statistically significant association was declared for variables with a *p*-value < 0.05. The result of this study was described in texts, tables, and other diagrammatic illustrations for clarity and better understandability.

### Result

#### Socio-demographic characteristics of participants

Of the total (399) selected samples, three hundred eighty-six participated in the study, with a response rate of 96.74%. Regarding the sex of the child, two hundred twelve (54.9%) were males and one hundred seventy-four (45.1%) were females. The mean age of the children was 25 months. Among the respondents, two hundred eight (53.9%) had a family size of five and above. The majority (69.4%) of the participants came from the urban area, while the rest came from the rural area. About one-fourth (25.9%) of mothers’ educational status was college and above, and nearly one-third (31.4%) of fathers attended college and above the level of education (Table 1).

**Table 1:** Sociodemographic characteristics of the participants in the pediatric ward of University of Gondar Comprehensive Specialized Hospital (*n* = 386)

Variable	Category	Frequency	Percent (%)
**Sex of child**	Male	212	54.9
	Female	174	45.1
**Age of child**	< 12 months	181	49.9
	12–24 months	73	19.2
	24–59 months	86	23.3
	> 59 months	45	11.7
**Family size**	< 5	178	46.1
	*≥* 5	208	53.9
**Place of residence**	Urban	268	69.4
	Rural	118	30.6
**Educationalstatusof**	Cannot read and write	91	23.6
**the mother**	Can read and write	16	4.1
	Primary school	71	18.4
	Secondary school	108	28
	College and above	100	25.9
**Educationalstatusof**	Cannot read and write	75	19.5
**the father**	Can read and write	28	7.3
	Primary school	49	12.7
	Secondary school	111	28.8
	College and above	122	31.4
**Religion**	Orthodox	328	85
	Muslim	53	13.7
	Protestant	5	1.3
**Occupation of mother**	Government employee	83	21.5
	Private employee	98	25.4
	Farmer	113	29.3
	Merchant	52	13.5
	Other	40	10.4
**Occupation of father**	Government employee	109	28.2
	Private employee	87	22.5
	Farmer	122	31.6
	Merchant	55	14.2
	Other	13	3.4

### Symptoms of hypoxemia in children

Among the total participants, almost all (97.7%) of the children had a history of coughing. Three hundred eighty-six (84.5%) of the children were able to breastfeed or drink. One hundred seventy (44.0%) of the children had difficulty breathing, and the majority (75.1%) of the children had a fever. Three hundred seventy-four (96.9%) of the children had fast breathing. One hundred fifty-two (39.4%) of the children had a history of fast breathing for 24 to 48 h (Table 2).

**Table 2:** Symptoms of hypoxemia among children with respiratory distress at the University of Gondar Comprehensive Specialized Hospital (*n* = 386)

Variable	Category	Frequency	Percent (%)
**Does the child able to drink/breastfeed?**	Yes	326	84.5
	No	60	15.5
**Does the child have a cough?**	Yes	377	97.7
	No	9	2.3
**How much duration?**	< 3 days	109	28.2
	3–5 days	184	47.7
	> 5 days	80	20.7
**Does the child have difficulty of**	Yes	170	44.0
**breath?**	No	216	56.0
**How much duration for difficulty of breathing?**	< 24 h.	89	53.6
	24–48 h.	66	39.8
	> 48 h.	11	6.6
**Does the child have a fever?**	Yes	290	75.1
	No	96	24.9
**Does the child have diarrhea?**	Yes	20	5.2
	No	366	94.8
**Does the child have vomiting?**	Yes	29	7.5
	No	357	92.5
**Does the child have abnormal body movement (seizure)?**	Yes	3	0.8
	No	383	99.2
**Does the child have fast breathing?**	Yes	374	96.9
	No	12	3.1
**How much duration has the child fast breathing?**	< 24 h.	82	21.2
	24–48 h.	152	39.4
	> 48 h.	143	37.0

### Clinical signs of hypoxemia on admission

Among the total number of cases that participated in this study, the majority (84.7%) had no cyanosis. Two hundred thirty-one (59.8% of them) were conscious. Almost all patients (99.2%) had nasal flaring; two hundred ninety-three (75.9%) of cases had to grunt. Three hundred fifty- six (92.2%) of cases had no pallor. The majority (79.3%) of them had intercostal retraction, and two hundred forty-eight (64.2%) of the cases had sub-coastal retraction. Two hundred forty-four (63.0%) had severe pneumonia. Two hundred eighty (72.5%) of the children who participated in this study had a history of crackle at admission in pediatric emergencies (Table 3).

**Table 3:** Clinical signs of hypoxemia on admission among children with respiratory distress at the University of Gondar Comprehensive Specialized Hospital (*n* = 386)

Variables	Category	Frequency	Percent (%)
**Cyanosis**	Yes	59	15.3
	No	327	84.7
**Mental status**	Conscious	231	59.8
	Irritable	107	27.7
	Lethargic	48	12.4
**Anthropometric measurement**	No malnutrition	339	87.8
	MAM	23	6
	SAM	24	6.2
**Pallor**	Yes	30	7.8
	No	356	92.2
**Grunting**	Yes	293	75.9
	No	93	24.1
**Nasal Flaring**	Yes	383	99.2
	No	3	0.8
**Wheezing**	Yes	114	29.5
	No	272	70.5
**Crackles**	Yes	280	72.5
	No	106	27.5
**Chest indrawing/Subcostal retraction.**	Yes	248	64.2
	No	138	35.8
**Inter-costal retractions**	Yes	305	79.3
	No	80	20.7
**Suprasternal retraction**	Yes	112	29
	No	174	71
**Stridor**	Yes	26	6.7
	No	260	93.3
**Head nodding**	Yes	169	43.8
	No	217	56.2
**Diagnosis of the child**	Severe pneumonia	244	63
	HAAD/Asthma	83	21.5
	Bronchiolitis	22	5.7
	Croup	12	3.1
	Others	25	6.5

### Prevalence of hypoxemia in children with respiratory distress

The prevalence of hypoxemia among children in the pediatric ward of the University of Gondar Comprehensive Specialized Hospital was 245 (63.5%) with a 95% CI (58.8–68.4%).

### Socio-demographic characteristics of the children in non-hypoxemic and hypoxemic cases

**Fig. 1:**
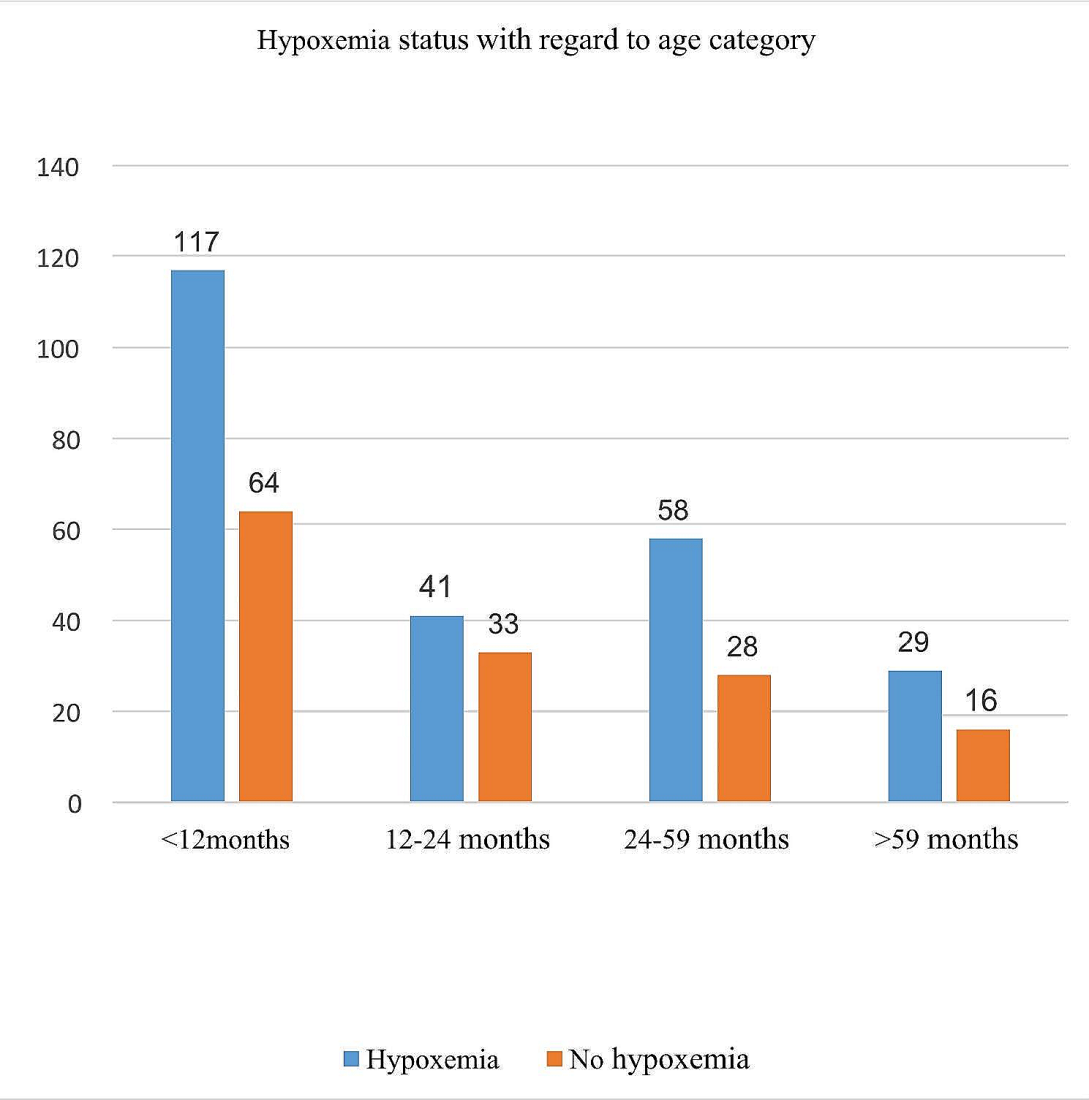
Status of hypoxemia among children with age category in the pediatric ward of University of Gondar Comprehensive Specialized Hospital (*n* = 386)

Among the total cases, one hundred forty-four (67.9%) males were hypoxemic, and seventy-six (43.6%) of the females were hypoxemic (*p*-value = 0.011). This study showed that most of the children (62.6%) who came from the urban area were hypoxemic, and sixty-eight (57.6%) of the children who came from the rural area were hypoxemic (*p* value = 0.494). One hundred seventeen (64.6%) of cases were hypoxemic in age less than twelve months from the total of one hundred eighty-one cases, and there was no significant association with hypoxemia (*P* = 0.831) (Table 4). Regarding the age group, one hundred seventy of the cases under the age of 12 months were hypoxemic, and fifty-eight of the cases were under the age category of 24–59 months and had hypoxemia (Fig. 1).

**Table 4:** Socio-demographic characteristics of the children in non-hypoxemic and hypoxemic children with respiratory distress at the University of Gondar Comprehensive Specialized Hospital (*n* = 386)

Variables		Total	Hypoxemic	Non- hypoxemic	*P*-Value
**Sex**	Male	212	144	65	0.011
	Female	174	76	98	
**Place of**	Urban	268	167	101	0.494
**residency**	Rural	118	68	40	
**Age of**	< 12months	181	117	64	
**child**	12-24months	74	41	33	
	24–59 months	86	58	28	0.831
	> 59 months	45	29	16	

### Clinical signs and symptoms in non-hypoxemic and hypoxemic cases

Cough was present in 98.0% of children in hypoxemic cases, but there was no statistical association between cough and hypoxemia (*p*-value = 0.729). Fast breathing was present in 98.4% of children in the hypoxemic group, and a statistical association was found between fast breathing and hypoxemia (*p*-value = 0.035). Breathing difficulty was found in 57.6% of hypoxemic cases, and a statistical association was found between the difficulty of breathing and hypoxemia (*p* value = < 0.001). Inability to feed (20.8%), impaired consciousness (24.9%), and cyanosis were significantly associated with hypoxemia (P = < 0.001). Chest indrawing, intercostal, and suprasternal retraction were present in hypoxemic children with 83.3%, 93.1%, and 43.7%, respectively, and there was a statistical association with hypoxemia (*p*-value = < 0.001) (Table 5).

**Table 5:** Clinical signs and symptoms in non-hypoxemic and hypoxemic children at the University of Gondar Comprehensive Specialized Hospital (*n* = 386)

Signs and Symptom	Hypoxemic (245)	Non-Hypoxemic (141)	*P* Value	OR
**Fever**	178(72.8%)	112(79.4%)	0.144	12.9
**Cough**	240(98.0%)	4(2.8%)	0.729	1.4
**Fast breathing**	241(98.4%)	133(94.3%)	0.035	0.73
**Breathing difficulty**	141(57.6%)	29(20.6%)	< 0.001	5.2
**Inability to feed**	51(20.8%)	7(4.9%)	< 0.001	0.19
**Stridor**	23(9.38%)	3(2.12%)	0.05	4.76
**Tachypnea** **RR** **≥** **50**	218(89.9%)	119(11.1%)	0.26	0.264
**Nasal flaring**	245(100%)	138(97.9)	0.048	
**Grunting**	179(73.1%	114(80.9%)	0.108	0.64
**Wheezing**	90(36.7%)	24(17.0%	< 0.001	2.83
**Crackle**	176(71.8%)	104(73.7%)	0.72	0.90
**Cyanosis**	57(23.3%)	2(1.4%)	< 0.001	21.07
**Pallor**	29(11.8%)	1(0.7%	0.07	17.8
**Impaired** **consciousness**	41(25.9%)	7(5.8%)	< 0.001	5.70
**Head nodding**	153(62.4%)	16(43.8%)	< 0.001	12.99
**Chest in drawing**	204(83.3%)	44(31.2%	< 0.001	11.24
**intercostal** **retractions**	228(93.1%)	78(55.3%)	< 0.001	10.83
**Suprasternal** **Retractions**	107(43.7%)	5(3.5%)	< 0.001	21.09

### Clinical predictors of hypoxemia according to sensitivity and specificity

For the clinical signs and symptoms, sensitivity, specificity, positive predictive value, and negative predictive value were carried out using statistical analysis, including the chi-square test. This study presented the clinical predictors of hypoxemia identified as the statistically significant predictor with a *p*-value < 0.05. The following variables were highly sensitive for hypoxemia: fast breathing with sensitivity (98.4%), nasal flaring (100.0%), chest indrawing (83.6%), and intercostal retraction (93.1%), but these variables were less specific to hypoxemia.

The most specific predictors of hypoxemia were breathing difficulty with specificity (79.4%), inability to feed (100.0%), wheezing (83.0%), cyanosis (98.6%), impaired consciousness (94.2%), head-nodding (88.7%), and suprasternal retraction (96.5%), but these variables were less sensitive to hypoxemia (Table 6).

**Table 6:** Clinical predictors of hypoxemia according to sensitivity and specificity of children with respiratory distress at University of Gondar Comprehensive Specialized Hospital (*n* = 386)

Variable	Sensitivity %	Specificity %	PPV %	NPV %
**Fever**	72.7	20.6	61.4	30.2
**Cough**	98.0	2.8	63.7	44.7
**Breathing difficulty**	57.6	79.4	82.9	51.9
**Fast breathing**	98.4	5.7	64.4	67.7
**Inability to feed**	20.8	100.0	87.9	36.5
**Stridor**	9.4	97.9	88.5	38.3
**Nasal flaring**	100.0	2.1	64.0	100.0
**Grunting**	73.1	19.1	61.1	29
**Wheezing**	36.7	83.0	78.9	43.0
**Crackle**	71.8	26.2	62.9	34.9
**Cyanosis**	23.3	98.6	96.6	42.5
**Impaired consciousness**	25.9	94.2	85.4	49.4
**Head nodding**	62.5	88.7	90.5	57.6
**Chest in drawing**	83.6	68.8	82.3	70.8
**Intercostal retractions**	93.1	44.7	74.5	78.8
**Suprasternal Retractions**	43.7	96.5	95.5	49.6
**Tachypnea** **> 50**	89.9	11.4%	64.7	55.1

PPV; Positive Predictive Value, NPV; Negative Predictive Value

### Multivariable logistic regression of the clinical signs and symptoms

After bivariable logistic regression analysis was carried out, the variables were included in multivariable logistic regression analysis to control potential confounders and identify a statistically significant association with hypoxemia. Inability to feed: those children able to feed were 87% times less likely to predict hypoxemia as compared to those unable to breastfeed, feed, or drink (AOR: 0.13, 95% CI: 0.02–0.77); head nodding: those children with head nodding were about 4 times more likely to predict hypoxemia (AOR: 4.1, 95% CI: 1.81–9.28); and chest indrawing: those children with the chest in drawing were about 3 times more likely to predict hypoxemia as compared to their counterparts (AOR: 3.08, 95% CI: 1.32–7.16) (Table 7).

**Table 7:** Multivariable logistic regression of the clinical sign and symptoms of children with respiratory distress at University of Gondar Comprehensive Specialized Hospital (*n* = 386)

Variable	Adjusted OR	95%CI	*P*-value
**Breathing difficulty**	1.16	0.47–2.87	0.740
**Inability to feed**	0.13	0.02–0.77	0.025*
**Grunting**	0.91	0.28–2.87	0.873
**Wheezing**	2.14	0.79–5.76	0.131
**Cyanosis**	4.93	0.53–15.87	0.161
**Impaired consciousness**	0.71	0.13–4.98	0.735
**Head nodding**	4.10	1.81–9.28	0.001*
**Chest in drawing**	3.08	1.32–7.16	0.009*
**Intercostal retractions**	2.44	0.80–7.36	0.113
**Suprasternal retractions**	4.10	0.92–18.16	0.063
**Tachypnea** **> 50**	0.87	0.38–1.98	0.754

*Statistically significant at *p*-value < 0.05

## Discussion

This study investigated the prevalence and clinical predictors of hypoxemia among children with respiratory distress at the University of Gondar Comprehensive Specialized Hospital, Northwest Ethiopia. In this study, the prevalence of hypoxemia among children in the pediatric emergency ward of the University of Gondar Comprehensive Specialized Hospital was 63.5% with a 95% CI (58.8 − 68.4%). This high prevalence of hypoxemia in children with respiratory distress becomes a public health concern and deteriorates the health status of children. In comparison with other studies, the prevalence of hypoxemia in this study was bad and higher, which needs reduction efforts. This finding was in line with the study conducted in Kenya (58.9%) [18].

The prevalence of hypoxemia in this study was higher than other studies conducted in Ethiopia (57.0%) [17], Colombia (47%) [19], Nepal (38.5%) [20], another study in Nepal (51%) [21], India (33.5%) [22] Peru (10%) [13], and the Netherlands (45%) [14]. This discrepancy might be due to variations in the health care system, variations in access to adequate healthcare services, health care facilities, and variations in the habit of proper hygiene and sanitation, which leads to respiratory tract infection, unclean water, inadequate access to food, which leads to immune suppression, and low levels of immunization coverage. As a result, children were easily infected and became hypoxemic due to late admission and inadequate care and treatment.

It might also be due to the variation in socio-demographic characteristics of the study population.

The clinical signs and symptoms of the patient with respiratory distress can predict hypoxemia.

This study presented the clinical predictors of hypoxemia in children diagnosed with respiratory distress. The most sensitive clinical signs and symptoms of hypoxemia were fast breathing with sensitivity (98.4%) and specificity (5.7%), nasal flaring sensitivity (100.0%) and specificity (2.1%), chest indrawing sensitivity (83.6%), and specificity (68.8%), and intercostal retraction sensitivity (93.1%) and specificity (44.7%). This finding showed that children with respiratory distress or respiratory difficulty who also have any of these predictors (fast breathing, chest indrawing, nasal flaring, and intercostal retractions) are more likely to be hypoxemic, and the patient needs oxygen supplementation in addition to other management measures, especially in health care settings where pulse oximetry is not available. So, these combined sensitive predictors of hypoxemia can help us easily diagnose hypoxemia, particularly in resource limited areas. This finding was supported by another study; the most sensitive predictors of hypoxemia were chest in drawing, while the most specific predictor of hypoxemia was the inability to breastfeed, feed, or drink [23]. In this study, the most sensitive predictor of hypoxemia was fast breathing, and the most specific predictor of hypoxemia was difficulty of breathing. But, in previous studies, the most sensitive predictor was chest wall indrawing, and the most specific predictor was the inability to feed. This discrepancy might be due to the variation in the health care setting, facility standards, and age of the sample population, in this study, the age of participants was wider. However, these variables are also important in our study. The most specific indicators of hypoxemia were breathing difficulty with specificity (79.4%) and sensitivity (57.6%), inability to feed specificity (100.0%) and sensitivity (20.8%), wheezing specificity (83.0%) and sensitivity (36.7%), cyanosis specificity (98.6%), impaired consciousness specificity (94.2%) and sensitivity (25.9%), head nodding specificity (88.7%) and sensitivity (62.5%), and supra-sternal retraction specificity (96.5%) and sensitivity (43.7%). This finding was supported by another study conducted in New York, USA [24]. Cyanosis, nasal flaring, chest indrawing, head nodding, inability to feed, and impaired consciousness were the clinical indicators of hypoxemia in children diagnosed with a respiratory infection. In another study in developing countries [25], the clinical indicators of hypoxemia were cyanosis and poor feeding, which are in agreement with this study. But cyanosis was not statistically significant with hypoxemia in our study. This might mean that the above clinical signs and symptoms of respiratory illness can easily help us indicate the presence of hypoxemia. But the percentage of sensitivity and specificity of the clinical predictors can vary. In this study, clinical signs like cyanosis, grunting, and nasal flaring were not significantly associated with hypoxemia. However, in another study (Nepal), cyanosis, grunting, and nasal flaring were significantly associated with hypoxemia, while in this study, cyanosis was a specific predictor of hypoxemia, and nasal flaring was a sensitive predictor of hypoxemia [26]. This discrepancy might be due to the difference in the management protocol of the health care stings and the variation in socio-economic and demographic factors.

Inability to feed: those children able to feed were 87% less likely to predict hypoxemia as compared to those unable to breastfeed, feed, or drink. This finding was supported by another study [24]. This might be because children who can feed might show less clinical signs and symptoms of respiratory distress, but if the child is unable to feed, the degree of hypoxemia is severe. The exact scientific reason for the inability to feed is that the protective factor of hypoxemia remains unknown; future researchers would do better to address this issue. Head nodding was one of the best clinical predictors of hypoxemia among children with respiratory distress; those children with head-nodding were about four times more likely to predict hypoxemia. This finding was supported by other studies [27]. This might be due to head- nodding being a severe sign of respiratory illness. The possible scientific reason for this situation is that patients with respiratory distress usually manifest head nodding due to the use of sternocleidomastoid and scalene muscle contractions during respiration. So, head nodding is an easily recognizable predictor of hypoxemia in children.

Chest indrawing was the other clinical predictor of hypoxemia in children diagnosed with respiratory distress; those children with the chest in drawing were about three times more likely to predict hypoxemia as compared to their counterparts. This finding was supported by another study [28]. This might be due to the patient being able to have a chest indrawing as a compensatory mechanism when they have significant respiratory distress. The possible justification for chest indrawing considered a clinical predictor of hypoxemia; there is scientific evidence that patients with respiratory distress will present with chest indrawing because patients in distress face a shortage of oxygen in the tissue as well as in the blood [29]. To compensate for this inadequacy of oxygen, the respiratory system uses the accessory muscles around the chest [30]. Due to the use of accessory muscles the patient will manifest chest indrawing. Therefore, health care professionals could take the clinical predictors as suggestive evidence in patients with respiratory distress for proper management and prevention of hypoxemia.

### Limitations of the study

In this study, some maternal related factors were not included as clinical predictors of hypoxemia.

We recommend future researchers conduct studies by including important maternal related variables.

## Conclusion

The prevalence of hypoxemia among children admitted to the pediatric ward was high. The statistically significant predictors of hypoxemia were the inability to feed, head nodding, and chest indrawing. Head nodding and chest indrawing were the risk factors of hypoxemia, while the inability to feed was the protective factor for hypoxemia. The most sensitive predictors of hypoxemia were fast breathing, nasal flaring, chest indrawing, and intercostal retraction. The best specific predictors of hypoxemia were breathing difficulty, inability to feed, wheezing, cyanosis, impaired consciousness, head nodding, and supra-sternal retraction. Therefore, medical attention should be given to children with chest indrawing and head nodding. The clinical predictors of respiratory distress identified in this study will be an input to developing intervention strategies for the prevention and management of hypoxemia.

### Recommendation

It is recommended to the health care settings to provide immediate special care for the children with head nodding and chest indrawing. The health policy makers better to focus on preventive measures, particularly for those patients with the statistically significant predictors and most specific clinical predictors. Future researchers also recommended to conduct studies including other maternal and child health related variables.

## Data Availability

The authors declared that the datasets used and analyzed in the current study are available upon reasonable request. The reader could contact the corresponding author for all data.

## Abbreviations

ALRTI: Acute Lower Respiratory Tract Infection
HAAD: Hyperactive Airway Disease
IMCI: Integrated Management of Childhood Illness
MOH: Ethiopian Ministry of Health
NPV: Negative Predictive Value
OR: Odds Ratio
Pao2: Partial Pressure of Oxygen
PPV: Positive Predictive Value
Spo2: Saturation of Oxygen by pulse oximetry
WHO: World Health Organization
